# Can Grasslands in Photovoltaic Parks Play a Role in Conserving Soil Arthropod Biodiversity?

**DOI:** 10.3390/life13071536

**Published:** 2023-07-10

**Authors:** Cristina Menta, Sara Remelli, Matteo Andreoni, Fabio Gatti, Valeria Sergi

**Affiliations:** 1 Department of Chemistry, Life Sciences and Environmental Sustainability, University of Parma, Viale delle Scienze 11/A, 43124 Parma, Italy; cristina.menta@unipr.it (C.M.); fabio.gatti@unipr.it (F.G.); 2 ESPERTA Benefit Corporation, Strada Giarola, 8, 43044 Collecchio, Italy; matteo.andreoni@studenti.unipr.it; 3 Department Civil, Environmental, Architectural Engineering and Mathematics (DICATAM), University of Brescia, via Branze 43, 25060 Brescia, Italy; sergi.valeria89@hotmail.it

**Keywords:** soil biodiversity conservation, soil fauna, soil organic matter

## Abstract

Under the increasing global energy demand, the new European Union Biodiversity Strategy for 2030 encourages combinations of energy production systems compatible with biodiversity conservation; however, in photovoltaic parks, panels shadowing the effects on soil health and biodiversity are still unknown. This study (location: Northern Italy) aimed to evaluate the effect of ground-mounted photovoltaic (GMPV) systems on soil arthropod biodiversity, considering two parks with different vegetation management: site 1—grassland mowed with tractor; site 2—grassland managed with sheep and donkeys. Three conditions were identified in each park: under photovoltaic panel (row), between the panel rows (inter-row), and around the photovoltaic plant (control). The soil pH and organic matter (SOM), soil arthropod community, biodiversity, and soil quality index (e.g., QBS-ar index) were characterised. Differences between the two GMPVs were mainly driven by the SOM content (higher values where grazing animals were present). No differences were observed in site 1, even if a high heterogeneity of results was observed for the soil biodiversity parameters under the panels. In site 2, SOM and pH, as well as arthropods biodiversity and QBS-ar, showed low values in the row. Soil fauna assemblages were also affected by ground-mounted panels, where Acarina, Collembola, Hymenoptera, and Hemiptera showed the lowest density in the row. This study suggests that ground-mounted solar panels had significant effects on below-ground soil fauna, and was more marked depending on the system management. Furthermore, the results obtained for the inter-row were similar to the control, suggesting that the area between the panel rows could be considered a good hotspot for soil biodiversity.

## 1. Introduction

In recent years, the increase in global energy demand and the need to replace fossil fuels, as one of the key drivers of climate change, have led to the development of renewable energy sources. In this context, the new European Union (EU) Biodiversity Strategy for 2030 promotes the development of economic models that are able to combine private and social-ecological interests in order to gain profit and, simultaneously, support ecosystem services, encouraging the combination of energy production systems compatible with biodiversity conservation [1]. The solar energy market has received extensive attention from governments worldwide, as the use of solar photovoltaic (PV) panels shows the greatest technical potential among the available renewable energy technologies [2]. However, despite its many advantages, the installation of these systems can have negative impacts on ecological and ecosystem services [3,4,5]. For example, it has been observed that site preparation for PV arrays often results in significant increases in onsite runoff and soil erosion [6]. Moreover, changes in the soil structure, vegetation cover, and albedo can raise air temperatures over PV arrays relative to the surrounding natural areas [7]. Finally, the realization of ground-based photovoltaic systems requires site modifications that may have various environmental impacts on landscapes and biodiversity, which are in contradiction with the goal of mitigating global warming [8].

Thus, designing and assessing large-scale PV systems combined with reducing the environmental impacts and the preserving soil biota ecosystem services will be essential to synergistically address ecological emergencies and socio-economic needs. 

Even if harmonization between energy production and safeguarding ecological processes have been investigated, the focus has been on ecosystem services underpinned by bird diversity, broadleaf plant diversity, wildflowers, and other species such as butterflies and bumblebees [8]; meanwhile, the effects on soil health and soil fauna biodiversity are still largely unknown. This gap in knowledge is extremely important considering that soil fauna can have substantial effects on soil health, optimizing ecosystem services beyond sustainable soil management, influencing the decomposition of organic matter, nutrient and water cycling, global carbon dynamics, and the suppression of soil-borne diseases and pests, in addition to improving soil quality [9]. In particular, soil microarthropods are found in many soils and are recognized as indicators of soil quality because they contribute to litter decomposition, nutrient cycling, and soil structure [10].

The installation of ground-mounted photovoltaic (GMPV) panels may induce direct effects on soil, modifying soil fertility, with a significant reduction in the water holding capacity and soil temperature, while the electrical conductivity (EC) and pH are increased [11,12]. Fluctuations in soil characteristics influence the abundance of microarthropods, which are concentrated in the litter layers and upper horizons of the soil and are susceptible to abiotic changes [13]. A study conducted by [14] in Chilean desert environments, with the purpose of evaluating the influence of solar power plants on microclimatic conditions and arthropod community collected by traps placed in the soil, showed that shady conditions can provide a refuge for some arthropod species. The authors of [15] discovered that the species richness of arthropods, collected by pitfall traps, differed between green roofs with and without photovoltaic panels, with 23% more morphospecies in the green roofs without photovoltaic panels. In addition, the study highlighted that distinct functional groups could respond differently to the presence of PV panels. Therefore, soil arthropods, as well as the ecosystem services related to them, may be altered by land use change induced by ground-mounted photovoltaic panels, so proper and focused investigations are required. 

Given this, we investigated the effects of two GMPV solar systems on soil arthropods, considering both the impact of panels shadowing and inter-row. Specifically, this study aims to (i) deepen the impact of panels on the soil chemical parameters (e.g., pH and soil organic matter (SOM)), (ii) investigate the effect of solar system management on arthropod biodiversity, (iii) highlight how solar panels affect the heterogeneity of arthropods community inside photovoltaic plants, and (iv) define which arthropods are the most affected by soil conditions created by the panels. 

This study aims to set the stage for considering soil fauna biodiversity as an important component to ensure the environmental sustainability of such structures and help meet policy planning criteria and environmental policy objectives.

## 2. Materials and Methods

### 2.1. Study Area and Soil Sampling

Two GMPV systems were identified as study areas: the first one located in Lombardy (Gusciana) and the second one in Emilia-Romagna (Sissa). The main characteristics of the two systems are summarized in Table 1.

Each photovoltaic system was sampled two times during the monitoring period (first week of June 2021 and first week of October 2021). Each time, 30 soil samples were collected by digging up 10 × 10 × 10 cm of soil using a spade: 10 under the panel (row), 10 between the photovoltaic panels (inter-row), and 10 in the area surrounding each system (control). A total of 120 soil samples were taken and transported to the laboratory for chemical analyses and for arthropod extraction and characterization. From the sampling to the extraction phase, the soil samples were kept undisturbed, protected from thermal and mechanical shocks, and placed in the extractor system within 24 h.

### 2.2. Chemical Analyses

The pH and soil organic matter (SOM) were detected for each soil sample (after arthropod extraction). Before carrying out the chemical analyses, air-dried soil samples were ground to pass a 2 mm sieve. The pH analysis was conducted on a soil−distilled water mixture (1:2.5 *w*/*v*) using a pH meter with automatic temperature compensation [16]. That is, for each soil sample, 10.0 g was weight and 25 mL of water was added. Then, the soil–water mixture was shaken thoroughly for 15 min with a shaking machine, and allowing the mixture stand for 30 min before taking the pH measurement. SOM was detected using LOI-Loss on Ignition, i.e., after initially oven drying 6 g of soil sample at 105 °C for 24 h, the samples were ignited in a muffle furnace for 4 h at 400 °C. LOI calculated the percentage of organic matter by comparing the weight of a sample before and after the soil was ignited [17].

### 2.3. Arthropods Extraction and Characterization

Soil arthropods were extracted from each soil sample using a Berlese–Tullgren funnel (time of extraction: 10 days; heat source 40 watts at 30 cm distance from soil sample, mesh size of 2 × 2 mm). The extracted specimens were collected and preserved in a solution of ethyl alcohol−glycerol (3:1) (all of the specimens of this study are deposited at the University of Parma). Specimens were identified at subclasses for Acarina and order level for other groups and were counted using a 8–50× stereomicroscope. Within holometabolous insects, adults and larvae were considered separately, because of their often-different trophic niches. For defining the soil biological quality, the QBS-ar index was applied [18]. The QBS-ar index (i.e., biological soil quality based on arthropods) evaluates the capacity of soil to host animals that are particularly sensitive to changes in the soil environment, due to their morphological characteristics, and they are thus more informative of soil quality variations than the more tolerant ones. QBS-ar, provides an indication on soil biological quality related to land degradation, and is based on the morphological features mentioned above, assigning an Eco-Morphological index (EMI), ranging between 1 and 20, in relation to the adaptation level to soil (1 = no adaptation; 20 = total adaptation) at each taxon. QBS-ar shows the results of the sum of each maximum EMI score assigned at each taxon identified in the soil sample (details of the method are described in [18,19]).

### 2.4. Statistical Analysis

Neither the chemical nor arthropod parameters met the assumptions for parametric tests; thus, non-parametric analyses were carried out. All of the calculations were made on raw data (ind./sample).

The factor analysis of mixed data (FAMD) was run on quantitative (pH, SOM, total abundance, number of taxa, and QBS-ar) and qualitative (season: Autumn/Spring, condition: Row/Inter-row/Control, and site: Gusciana/Sissa) variables to understand which data contributed most to overall variability.

FAMD data were computed and presented using the FactoMineR (version 2.4) and factoextra (version 1.0.7) packages, respectively.

The Kendall rank correlation coefficient between the chemical parameters was calculated.

The Kruskal–Wallis test was performed for multiple comparisons, followed by the Wilcoxon test to compare the row and inter-row (paired data), and the Mann–Whitney to compare the control and inter-row/row (independent samples).

For the arthropod assemblage analysis, the community matrix was square-root transformed to reduce the relative influence of the most frequent orders, then non-metric multidimensional scaling (NMDS), based on the Bray−Curtis dissimilarity index, followed by a permutational multivariate analysis of variance (PERMANOVA), was performed to visualize how the patterns above illustrated the influence of the grouping of arthropods communities. Finally, an analysis of similarity percentages (SIMPER) was used to test which arthropod groups were driving the differences in the community assemblages.

Ordination, PERMANOVA, and SIMPER were performed with the vegan package, with pairwiseAdonis package as post hoc for PERMANOVA. 

The Generalized Linear Mixed-Effects Model was applied on arthropod parameters and on the single orders (only those accounting for a cumulative dissimilarity between communities of 60%, resulting from SIMPER analysis), considering quantitative and qualitative variables selected with FAMD as the fixed effects, and the existence of a pairing between Row and Inter-row as a random effect. 

Orders that were associated with a particular season, condition, or site, and the statistical significance of the association, were determined using a permutation test with the multipatt function from the indicspecies package (version 1.7.12). 

All of the analyses mentioned above were applied to the GMPV systems data, while for the preliminary data obtained in the APV system, only multiple and pairwise comparisons between conditions on the chemical data and soil arthropods parameters (total abundance, number of taxa, and QBS-ar) were applied. 

A *p*-value ≤ 0.05 was considered significant. All of the analyses were performed using R (v.4.0.5) [20].

## 3. Results

The environmental variables considered were the site and the condition to which the samples belonged, as well as the sampling season. However, because of the low contribution of seasonality to the overall data variability (Figure S1), season was not considered in the analysis of the chemical (SOM and pH) and arthropod (number of groups, total abundance, and QBS-ar) parameters.

### 3.1. Chemical Parameters

Both SOM and pH differed depending on the photovoltaic system, and were higher at Gusciana (*p* < 0.001, both; Table 2).

Moreover, no differences between conditions were observed within the Sissa system, unlike Gusciana (*p* < 0.01, for both SOM and pH). SOM was higher for the control than the rows under the panels and in the inter-row (*p* ≤ 0.01 and *p* ≤ 0.001, respectively); while pH was more alkaline in inter-row than in the row and control (*p* < 0.01, both).

SOM was negatively correlated with pH (τ = −0.54, *p* < 0.001), so in the following models, only one chemical parameter was considered.

### 3.2. Arthropod Community

A total of 24 taxa, with a density of 315.4 ind./m^2^, were extracted (Tables S1 and S2), with most of them belonging to Acarina (55%), Collembola (21%), and Hymenoptera (13%). Within the remaining groups, only Hemiptera (4%), Isopoda (2%), Coleoptera (larvae), and Thysanoptera (1%, both) accounted for at least 1% of the specimens extracted. 

**Figure 1:**
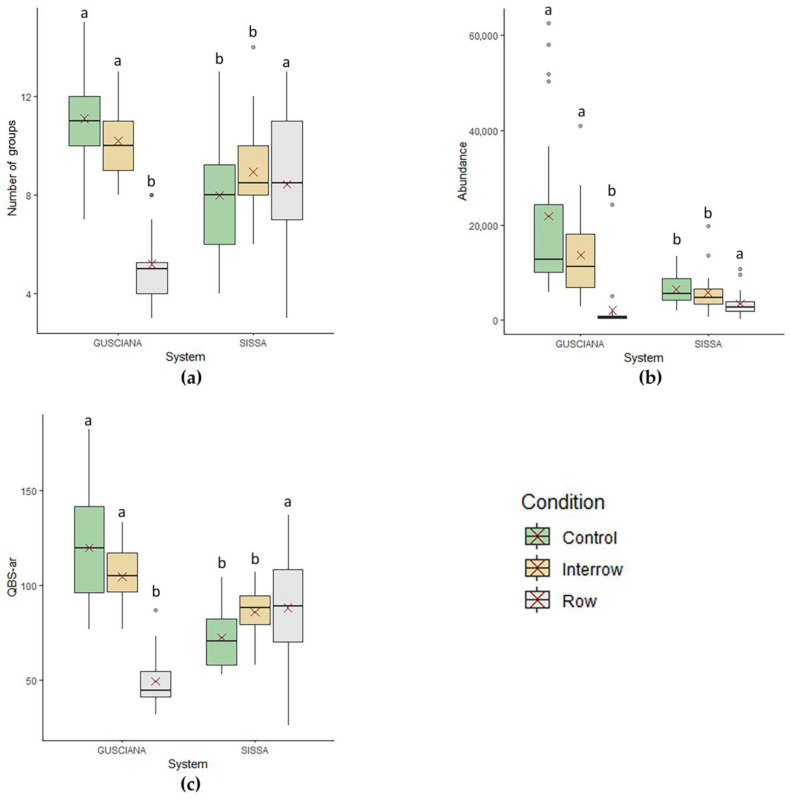
Boxplots of (**a**) number of taxa, (**b**) total abundance (ind./m^2^), and (**c**) QBS-ar value under each condition in the two GMPV systems. The bottom and top of each box represent the lower and upper quartiles, respectively; the line inside each box shows the median, the red cross shows the mean, and the whiskers indicate minimal and maximum observations. Different letters above the box of the same condition mean a significant difference (*p* ≤ 0.05) between the two systems.

The number of groups found in the samples were dependent only on the conditions under which the sample was collected (*p* < 0.001; Figure 1a); with generally lower values in the row than in the inter-row and control (*p* < 0.001, both). 

Both abundance and QBS-ar values depended on the SOM (estimates: abundance = 16.05 and QBS-ar = 1.53; *p* < 0.001, both), photovoltaic system (*p* ≤ 0.05, with both abundance and QBS-ar having higher values in Gusciana), and condition (*p* < 0.001, both), with generally lower values in the row that in the inter-row and control (*p* < 0.001, both) (Figure 1b,c). However, within Sissa, the QBS-ar values were lower in the control than in the row and inter-row (*p* ≤ 0.01, both; Figure 1c).

**Figure 2:**
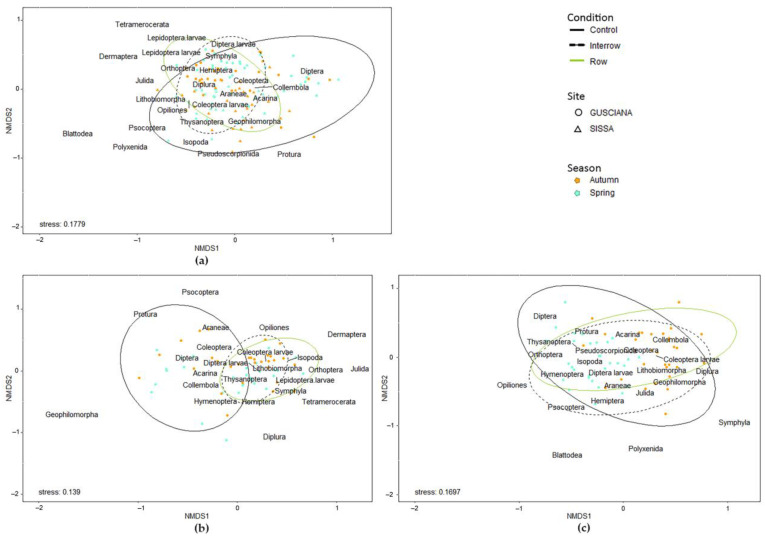
Bray–Curtis-based NMDS plot of arthropod community composition under the three conditions in the two seasons in (**a**) both GMPV systems, (**b**) Gusciana, and (**c**) Sissa. Points represent samples.

Arthropod assemblages were affected by the sampling season, photovoltaic system, and condition (*p* ≤ 0.001, all) (Figure 2a). Indeed, within photovoltaic systems (Figure 2b,c), differences depended on the season (*p* < 0.01, both) and condition (*p* < 0.01 and *p* ≤ 0.001, Sissa and Gusciana, respectively), with a significant effect of SOM found in the Gusciana system.

Acarina, Collembola, Hymenoptera, and Hemiptera are the orders accounting for most of the dissimilarities (Table 3).

Acarina and Collembola had a similar distribution, both influenced by SOM (estimate: Acarina = 6.73, Collembola = 12.98; *p* < 0.001, both), season (*p* < 0.001, both higher in Autumn), photovoltaic system (*p* < 0.01 and *p* < 0.001, respectively; both higher in Gusciana), and condition (*p* < 0.001), with both being lower in the row than in the inter-row and control (*p* < 0.001, all).

Hymenoptera and Hemiptera were both influenced by SOM (estimate: Hymenoptera = 16.82, Hemiptera = 15.17; *p* < 0.001, both), season (*p* < 0.001, both higher in Spring), and condition (*p* < 0.001), with both being lower in the row than in the inter-row and control (*p* < 0.001, all); however, no differences were observed between the two photovoltaic systems.

Some orders were associated with a particular season, condition, or site (Table 4).

## 4. Discussion

It is known that the presence of PV systems significantly alters the soil surface, reduces the surface albedo, changes the precipitation distribution, and forms a heat island effect [21]. These changes critically impact the driving factors of the local microclimate, so that soils and organisms below panels are subjected to variable light gradients that alter the evaporation and soil water retention, soil moisture, and soil temperature, on both temporal and spatial scales [5,21,22,23]. Under a Mediterranean climate, the changes in microclimate under the solar panels may be higher, with solar panels that can reduce the soil temperature in spring and in summer by about 2–5 °C [11,24]. In addition, shifts in rainfall can have a greater ecological impact than shifts in temperature [25,26,27]. This study aimed to investigate the effect of ground-mounted photovoltaic panels on the soil arthropod community, both directly and indirectly (through changes in soil properties), as well as considering the role of system management, in order to identify the best practices to preserve soil quality in photovoltaic systems. 

In this study, a negative correlation between pH and SOM was observed. Considering the previous use of the study areas, those results were in accordance with some studies [28,29] highlighting that in soils subjected to agricultural practices and poultry farming, there could be an increase in SOM related to more acidic soils. However, very little difference in pH and organic matter has been reported between the soil in the panel row and the undisturbed area around the plant. Conversely, in this study, the pH in the Gusciana inter-row was higher compared with the other conditions, supporting [12] who observed that 7 years after the installation of a power plant in Viterbo Province, the soil pH was increased in the inter-row. Moreover, SOM resulted in higher control conditions in Gusciana, maybe as a result of using grazing animals for vegetation management; indeed, donkey manure is very high in organic matter. A higher organic matter content also affects the arthropod abundance and QBS-ar value, as previously observed by [30], so this could be the reason for those parameters being higher in Gusciana. Even if a generalization is possible, under the panels, all parameters tend to be lower, while outside them, arthropod community patterns and soil biological quality are strongly dependent on system management. It is important to outline that, while arthropod parameters outside the panels benefit from the Gusciana management system, likely due to the high content of SOM together with the absence of disturbing factors such as passing tractors, under the panel, soil arthropods are favoured in the Sissa system. It should be noted that, in the Gusciana system, grazing animals tend to rest and seek shelter under the panels, and this behaviour could be the explanation for the differences in soil arthropod presence under panels between the two systems; indeed, animals resting represent a disturbing factor for soil fauna as it reduces soil cover, leads to soil compaction, and increases erosion, thus inducing a loss of soil invertebrates [31,32]. Another possible explanation is that Sissa panels are smaller, cover less soil surface, and have a wider inter-row than the Gusciana ones. This could have led to an interchange between the soil arthropod community under the panels and in the inter-row, which could have act as a biodiversity hotspot, so that the two communities resulted in being more similar in Sissa than in Gusciana. This was confirmed by the community structure, which was different under the panels than outside them, but with a more marked dissimilarity in the Gusciana system. 

In this study, Acarina, Collembola, Hymenoptera, and Hemiptera were the taxa that drove the major differences between the arthropod communities, with all of them being influenced by the SOM content. These taxa were affected by seasonality, with Acarina and Collembola being higher in Autumn, and Hymenoptera and Hemiptera having increased densities in Spring. However, all of them were negatively affected by the presence of panels, while the inter-row showed favourable conditions similar to the control, with almost the same arthropod communities. On the contrary, Pseudoscorpionidae were found to be particularly linked to the under-panel (row) condition. Pseudoscorpionidae are carnivorous arthropods and important predators that often co-occur with Psocoptera [10,33]; in this study, they were found only in Sissa, where they likely had better conditions under the panels, where the soil moisture was often higher and competitors for food resources (e.g., Geophilomorpha) were scarce [34]. The larvae of Diptera and Coleoptera, on the other hand, are often associated with higher soil temperatures and in soils rich in organic matter [33,35], and in this study, they were more linked to soils outside the panels (mostly in Gusciana). This is in accordance with [26,34], who found a lower temperature under solar panels to be a direct effect of shading. In effect, photovoltaic panels have a warming effect on the soil temperature in winter and a cooling effect on soils in the other seasons [2]. Solar panels also intercept precipitation, and [26] found a significant reduction in soil humidity under solar panels in the Mojave Desert. Heterogeneity in under-panel microclimatic conditions [26] could be the cause of the observed heterogeneity in the soil arthropod community composition. In addition to differences between arthropod communities due to system management, and thus soil organic matter content, an effect of seasonality was also observed. This effect was only perceived on the community structure and not the overall arthropod parameters (e.g., total abundance, diversity, or QBS-ar). Different sampling times can be characterized by different climatic conditions that, by altering the soil microclimate and indirectly modifying resource availability and food web composition [36,37], affect the arthropod community structure [38]. Other studies have shown that sometimes, the main predictor for arthropod community is the sampling date, because of the greater vegetation biomass present in autumn and the consequently greater resources for microarthropods, combined with favourable pedo-climatic conditions [38,39]. They found, however, that Acarina were more abundant during warm-dry conditions and Collembola were more abundant during cold-wet conditions; on the contrary, in this study, the same increasing pattern was observed in Autumn for both taxa.

Broadly, this study suggests that ground-mounted solar panels had significant effects on below-ground soil fauna, mainly depending on PV system management; however, where those effects were not evident, it was because a high heterogeneity of responses was observed, probably due to the non-uniform solar radiation and water availability [27].

## 5. Conclusions

The understanding of the impacts of photovoltaic systems on the soil environment is emerging, and knowledge about changes to the microclimate, soil (carbon cycling, soil microbial community composition, and soil moisture), and vegetation is being updated; however, the effects on soil arthropods biodiversity are still unknown. This study represents a first attempt to understand how photovoltaic systems could affect soil conditions and edaphic fauna biodiversity. The results obtained highlight that soil arthropods could be affected by the presence of ground-mounted panels, but with an impact that largely depends on park management. At the same time, the areas between the panels were similar to the control ones, and could thus represent biodiversity conservation hotspots.

## Figures and Tables

**Table 1:** The main characteristics of the two GMPV study areas.

	GUSCIANA (BS)	SISSA (PR)
Region	Lombardy	Emilia-Romagna
Year of completion	2010	2012
Power (kWp)	5525	2088
Panel modules material	polycrystalline silicon	polycrystalline silicon
Size of the panels (m)	0.99 × 1.64	0.99 × 1.61
Surface covered by panels (m^2^)	39,000	32,630
Ground clearance (m)	0.5–3	0.5–2.4
Distance between panels (m)	3.5	4
Former destination use of the area	Poultry farming with a building made from Eternit	Agricultural
Vegetation management	By animals (donkeys or sheep), trimmer, or a forage harvester on a small excavator	Tractor or forage harvester
Panels’ washing	3 times from March to September without using detergent	no

**Table 2:** Mean ± st.err. of pH and soil organic matter (SOM) content in the two GMPV systems. ^a,b^ Different uppercase letters indicate a statistically significant difference (*p* ≤ 0.05) between conditions.

	Gusciana			Sissa		
	Control	Inter-Row	Row	Control	Inter-Row	Row
pH	7.40 ± 0.14 ^b^	7.85 ± 0.05 ^a^	7.54 ± 0.09 ^b^	7.86 ± 0.03	7.84 ± 0.02	7.87 ± 0.01
SOM (%)	13.12 ± 0.92 ^a^	9.08 ± 0.59 ^b^	10.04 ± 0.41 ^b^	7.70 ± 0.36	8.32 ± 0.41	7.78 ± 0.23

**Table 3:** The results of the SIMPER analysis for contrasts within seasons, conditions, and photovoltaic systems. Most influential arthropod groups accounting for a cumulative dissimilarity of 70% are shown. Overall (%): average contrasts dissimilarity; Cum. (%): ordered cumulative contribution of each arthropod group. Asterisks mean significant differences in the community matrix between contrasts: * *p* < 0.005 and ** *p*< 0.01.

Parameter	Contrasts			Overall %	Most Influential Groups	Cum. %
Season	Autumn	-	Spring	51 *	Acarina	0.24
					Collembola	0.44
					Hymenoptera	0.57
					Hemiptera	0.65
					Diptera	0.69
					Isopoda	0.73
Condition	Row Row	-	Control	58 **	Acarina	0.29
					Collembola	0.48
					Hymenoptera	0.61
					Hemiptera	0.68
					Isopoda	0.72
		-	Inter-row	56 **	Acarina	0.24
					Collembola	0.45
					Hymenoptera	0.57
					Hemiptera	0.66
					Coleoptera larvae	0.71
	Inter-row	-	Control	40	Acarina	0.21
					Collembola	0.42
					Hymenoptera	0.56
					Hemiptera	0.65
					Diptera larvae	0.69
					Isopoda	0.72
Photovoltaic system	Sissa	-	Gusciana	51 *	Acarina	0.24
					Collembola	0.45
					Hymenoptera	0.57
					Hemiptera	0.64
					Isopoda	0.69
					Diptera	0.73

**Table 4:** Orders significantly associated with a particular season, condition, or site. Asterisks mean the significance of the association: * *p* ≤ 0.05, ** *p* ≤ 0.01, and *** *p* ≤ 0.001.

Parameter	Order
Season	
Spring	Isopoda **
	Lepidoptera larvae *
	Orthoptera **
	Psocoptera **
Autumn	Diplura **
	Hymenoptera larvae *
	Lithobiomorpha **
Condition	
Row	Pseudoscorpionida **
Inter-row	Geophilomorpha *
Control + Inter-row	Coleoptera larvae ***
	Diptera larvae ***
	Hemiptera ***
	Hymenoptera ***
	Hymenoptera larvae **
	Lepidoptera larvae *
	Symphyla **
Photovoltaic system	
Sissa	Diplura *
	Isopoda ***
	Pseudoscorpionida **
	Psocoptera **
	Thysanoptera ***
Gusciana	Diptera larvae ***
	Hymenoptera larvae ***
	Lepidoptera larvae ***
	Symphyla ***

## Data Availability

Data are contained within the article or Supplementary Material. The data presented in this study are available upon Supplementary Material.

## Supplementary Materials

The following supporting information can be downloaded at: https://www.mdpi.com/article/10.3390/life13071536/s1, Figure S1: FAMD output; Table S1: Abundance of arthropods groups in each condition, at (a) Spring and (b) Autumn, within Gusciana GMPV system; Table S2: Abundance of arthropods groups in each condition, at (a) Spring and (b) Autumn, within Sissa GMPV system.

## References

[1] European Commission Committee, Committee of the Regions EU (2020). Biodiversity Strategy for 2030 Bringing Nature Back into Our Lives.

[2] Yue S., Guo M., Zou P., Wu W., Zhou X. (2021). Effects of photovoltaic panels on soil temperature and moisture in desert areas. Environ. Sci. Pollut. Res..

[3] Weselek A., Ehmann A., Zikeli S., Lewandowski I., Schindele S., Högy P. (2019). Agrophotovoltaic systems: Applications, challenges, and opportunities. A review. Agron. Sustain. Dev..

[4] Hernandez R.R., Armstrong A., Burney J., Ryan G., Moore-O’leary K., Diédhiou I., Grodsky S.M., Saul-Gershenz L., Davis R., Macknick J. (2019). Techno–ecological synergies of solar energy for global sustainability. Nat. Sustain..

[5] Hernandez R.R., Easter S.B., Murphy-Mariscal M.L., Maestre F.T., Tavassoli M., Allen E.B., Barrows C.W., Belnap J., Ochoa-Hueso R., Ravi S. (2014). Environmental impacts of utility-scale solar energy. Renew. Sustain. Energy Rev..

[6] Cook P. (2011). Infrastructure, rural electrification and development. Energy Sustain. Dev..

[7] Barron-Gafford G.A., Minor R.L., Allen N.A., Cronin A.D., Brooks A.E., Pavao-Zuckerman M.A. (2016). The Photovoltaic Heat Island Effect: Larger solar power plants increase local temperatures. Sci. Rep..

[8] Semeraro T., Pomes A., Del Giudice C., Negro D., Aretano R. (2018). Planning ground based utility scale solar energy as green infrastructure to enhance ecosystem services. Energy Policy.

[9] Veen G.F., Jasper Wubs E.R., Bardgett R.D., Barrios E., Bradford M.A., Carvalho S., De Deyn G.B., de Vries F.T., Giller K.E., Kleijn D. (2019). Applying the Aboveground-Belowground Interaction Concept in Agriculture: Spatio-Temporal Scales Matter. Front. Ecol. Evol..

[10] Menta C., Remelli S. (2020). Soil Health and Arthropods: From Complex System to Worthwhile Investigation. Insects.

[11] Armstrong A., Ostle N.J., Whitaker J. (2016). Solar park microclimate and vegetation management effects on grassland carbon cycling. Environ. Res. Lett..

[12] Moscatelli M.C., Marabottini R., Massaccesi L., Marinari S. (2022). Soil properties changes after seven years of ground mounted photovoltaic panels in Central Italy coastal area. Geoderma Reg..

[13] Chikoski J.M., Ferguson S.H., Meyer L. (2006). Effects of water addition on soil arthropods and soil characteristics in a precipitation-limited environment. Acta Oecologica.

[14] Suuronen A., Muñoz-Escobar C., Lensu A., Kuitunen M., Celis N.G., Astudillo P.E., Ferrú M., Taucare-Ríos A., Miranda M., Kukkonen J.V.K. (2017). The Influence of Solar Power Plants on Microclimatic Conditions and the Biotic Community in Chilean Desert Environments. Environ. Manag..

[15] Schindler B.Y., Blaustein L., Lotan R., Shalom H., Kadas G.J., Seifan M. (2018). Green roof and photovoltaic panel integration: Effects on plant and arthropod diversity and electricity production. J. Environ. Manag..

[16] Edagricole, Società Italiana della Scienza del Suolo (1985). Metodi Normalizzati di Analisi del Suolo.

[17] Miano T., Mondelli D., Claudio C., Miano T., Associazione Italiana dei Laboratori Pubblici di Agrochimica, Società Italiana della Scienza del Suolo (2015). Sostanza Organica e Carbonio Organico. Metodi di Analisi Chimica del Suolo.

[18] Parisi V., Menta C., Gardi C., Jacomini C., Mozzanica E. (2005). Microarthropod communities as a tool to assess soil quality and biodiversity: A new approach in Italy. Agric. Ecosyst. Environ..

[19] Menta C., Conti F.D., Pinto S., Bodini A. (2018). Soil Biological Quality index (QBS-ar): 15 years of application at global scale. Ecol. Indic..

[20] R Core Team (2021). R: A Language and Environment for Statistical Computing.

[21] Luo L., Zhuang Y., Liu H., Zhao W., Chen J., Du W., Gao X. (2023). Environmental impacts of photovoltaic power plants in northwest China. Sustain. Energy Technol. Assessments.

[22] Hernandez R.R., Hoffacker M.K., Murphy-Mariscal M.L., Wu G.C., Allen M.F. (2015). Solar energy development impacts on land cover change and protected areas. Proc. Natl. Acad. Sci. USA.

[23] Armstrong A., Waldron S., Whitaker J., Ostle N.J. (2014). Wind farm and solar park effects on plant-soil carbon cycling: Uncertain impacts of changes in ground-level microclimate. Glob. Chang. Biol..

[24] González-Ubierna S., Lai R. (2019). Modelling the effects of climate factors on soil respiration across Mediterranean ecosystems. J. Arid. Environ..

[25] Thorne J.H., Boynton R.M., Flint L.E., Flint A.L. (2015). The magnitude and spatial patterns of historical and future hydrologic change in California’s watersheds. Ecosphere.

[26] Tanner K.E., Moore-O’Leary K.A., Parker I.M., Pavlik B.M., Hernandez R.R. (2020). Simulated solar panels create altered microhabitats in desert landforms. Ecosphere.

[27] Holloway L., Starry O., McClung R., Rosenstiel T., Portland State University (2020). The influence of microclimates created by photovoltaic panels and irrigation on green roof ecosystem properties. J. Living Arch..

[28] Sharma S.B. (2022). Trend setting impacts of organic matter on soil physico-chemical properties in traditional vis -a- vis chemical-based amendment practices. PLoS Sustain. Transform..

[29] Hilimire K., Gliessman S.R., Muramoto J. (2012). Soil fertility and crop growth under poultry/crop integration. Renew. Agric. Food Syst..

[30] Begum F., Bajracharya R.M., Sharma S., Sitaula B.K. (2010). Influence of slope aspect on soil physico-chemical and biological properties in the mid hills of central Nepal. Int. J. Sustain. Dev. World Ecol..

[31] Eldridge D.J., Delgado-Baquerizo M., Travers S.K., Val J., Oliver I. (2016). Do grazing intensity and herbivore type affect soil health? Insights from a semi-arid productivity gradient. J. Appl. Ecol..

[32] Freiberg J.A., Dambros C.D.S., Rodrigues E.N.L., Teixeira R.A., Vieira Â.D.H.N., de Almeida H.S., Carvalho P.C.D.F., Jacques R.J.S. (2019). Increased grazing intensity in pastures reduces the abundance and richness of ground spiders in an integrated crop-livestock system. Agron. Sustain. Dev..

[33] Ghiglieno I., Simonetto A., Orlando F., Donna P., Tonni M., Valenti L., Gilioli G. (2020). Response of the Arthropod Community to Soil Characteristics and Management in the Franciacorta Viticultural Area (Lombardy, Italy). Agronomy.

[34] Lambert Q., Bischoff A., Cueff S., Cluchier A., Gros R. (2021). Effects of solar park construction and solar panels on soil quality, microclimate, CO _2_ effluxes, and vegetation under a Mediterranean climate. Land Degrad. Dev..

[35] McColloch J.W., Hayes W.P. (1922). The Reciprocal Relation of Soil and Insects. Ecology.

[36] Block W. (1991). To Freeze or Not to Freeze? Invertebrate Survival of Sub-Zero Temperatures. Funct. Ecol..

[37] Frampton G.K., Van Den Brink P.J., Gould P.J.L. (2000). Effects of spring precipitation on a temperate arable collembolan community analysed using Principal Response Curves. Appl. Soil Ecol..

[38] Santorufo L., Van Gestel C.A.M., Maisto G. (2014). Sampling season affects conclusions on soil arthropod community structure responses to metal pollution in Mediterranean urban soils. Geoderma.

[39] Hopkin S.P. (1997). Biology of the Springtails: (Insecta: Collembola).

