# Supplementary material for: Can Grasslands in Photovoltaic Parks Play a Role in Conserving Soil Arthropod Biodiversity?
Source: Life (Basel). 2023 Jul 10;13(7):1536. doi: 10.3390/life13071536 (PMC10381872; doi:10.3390/life13071536)
Supplement: Supplementary file 1 [file life-13-01536-s001.zip › life-2456386-supplementary.pdf]

Supplemental materials

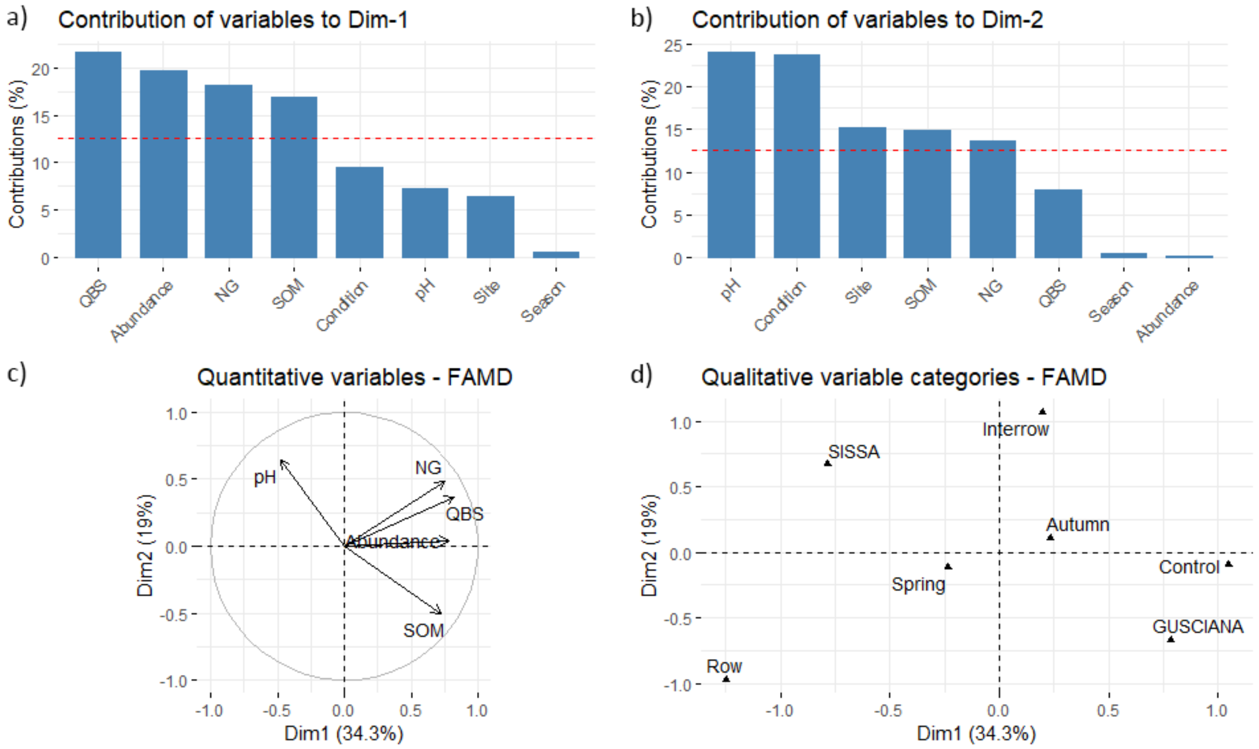

Figure S1. FAMD output. **(a)** and **(b)** show the contribution of variables to the dimensions 1 and 2 respectively. The red dashed line indicates the expected average value if the contributions were uniform. In **(c)** and **(d)** the results for quantitative and qualitative variables are shown respectively.

Table S1. Abundance of arthropods groups in each condition, at **(a)** Spring and **(b)** Autumn, within Gusciana GMPV system.

**(a)**		Row										Interrow										Control									
		I	II	III	IV	V	VI	VII	VIII	IX	X	I	II	III	IV	V	VI	VII	VIII	IX	X	I	II	III	IV	V	VI	VII	VIII	IX	X
Arachnida	Acarina	10	3	0	11	8	2	25	7	3	3	125	40	132	89	175	229	165	152	102	80	146	217	208	125	130	371	147	181	289	188
	Araneae	0	0	0	0	0	2	0	0	0	0	0	0	0	0	0	1	0	0	0	0	0	0	0	0	0	0	2	1	0	0
	Opiliones	0	0	0	0	0	0	0	0	0	0	0	0	0	0	0	0	0	0	0	0	0	0	0	0	0	0	0	0	0	0
	Pseudoscorpionida	0	0	0	0	0	0	0	0	0	0	0	0	0	0	0	0	0	0	0	0	0	0	0	0	0	0	0	0	0	0
Chilopoda	Geophilomorpha	0	0	0	0	0	0	1	0	0	0	0	0	0	0	0	0	0	0	0	0	0	0	0	0	0	0	0	0	0	0
	Lithobiomorpha	0	0	0	0	0	0	0	0	0	0	0	0	0	0	0	0	1	0	0	0	0	0	0	1	0	5	1	1	0	0
Diplopoda	Julida	0	0	0	0	0	0	0	0	0	0	0	0	0	0	0	0	0	0	0	0	0	0	0	0	0	0	0	3	0	0
	Polixenida	0	0	0	0	0	0	0	0	0	0	0	0	0	0	0	0	0	0	0	0	0	0	0	0	0	0	0	0	0	0
Pauropoda	Tetramerocerata	0	0	0	0	0	0	0	0	0	0	0	0	0	0	0	0	0	0	0	0	0	0	0	0	0	0	0	0	0	1
Symphyla	Symphyla	0	0	0	0	0	0	0	0	1	0	5	1	1	0	1	0	1	1	0	0	1	4	0	0	0	2	0	5	0	1
Malacostraca	Isopoda	0	0	0	0	0	0	0	0	0	0	0	0	0	0	0	5	0	0	0	0	0	0	0	0	2	0	0	0	0	0
Entognatha	Collembola	10	14	8	8	5	14	2	27	12	12	131	80	256	140	445	144	406	56	16	79	120	125	57	51	79	237	289	367	39	72
	Diplura	0	0	0	0	0	0	0	0	1	0	0	0	0	0	0	0	0	1	0	0	0	0	1	0	0	0	0	0	0	0
	Protura	0	0	0	0	0	0	0	0	0	0	0	0	0	0	0	0	0	0	0	0	0	0	0	0	0	0	0	0	0	0
Insecta	Blattodea	0	0	0	0	0	0	0	0	0	0	0	0	0	0	0	0	0	0	0	0	0	0	0	0	0	0	0	0	0	0
	Coleoptera	1	5	1	0	0	0	0	1	0	1	4	1	2	0	0	0	0	1	0	0	0	1	1	1	0	2	0	0	2	0
	Coleoptera larvae	0	0	1	0	0	0	0	0	0	1	3	3	3	4	1	4	3	12	4	3	0	1	3	1	0	12	1	3	1	2
	Dermaptera	0	0	0	0	0	0	0	0	0	0	0	0	0	0	0	0	0	0	0	0	0	0	0	0	0	0	0	0	1	0
	Diptera	8	4	13	3	0	5	3	3	0	1	2	5	9	4	6	4	7	1	4	3	2	8	8	3	2	4	0	0	1	0
	Diptera larvae	0	0	0	0	0	0	0	3	0	0	7	4	8	5	2	0	4	7	14	1	13	3	22	25	0	8	1	0	1	3
	Hemiptera	0	0	0	0	2	0	0	0	0	0	35	0	1	80	49	4	10	33	18	2	9	26	11	20	43	35	50	5	5	22
	Hymenoptera	3	4	5	1	4	0	2	0	5	0	2	2	6	32	20	2	38	22	53	7	80	222	14	2716	18	28	49	15	1	104
	Hymenoptera larvae	0	0	0	0	0	0	0	0	0	0	0	0	0	0	1	0	0	0	1	4	0	0	0	0	0	0	0	6	1	1
	Lepidoptera larvae	0	0	0	0	0	0	0	0	0	0	1	1	1	1	0	1	0	1	0	0	0	0	5	0	0	2	1	0	0	1
	Orthoptera	0	0	0	0	0	0	0	0	0	0	0	0	0	0	1	1	0	0	0	0	0	0	1	0	1	4	0	1	1	0
	Psocoptera	0	0	0	0	0	0	0	0	0	0	0	0	0	0	0	0	0	0	0	0	0	0	0	0	0	0	0	0	0	0
	Thysanoptera	0	0	0	0	0	0	0	0	0	0	0	2	0	0	0	1	1	0	0	0	0	1	0	0	3	0	0	0	2	0
	N° of groups	5	5	5	4	4	4	5	5	5	5	10	10	10	8	10	11	10	11	8	8	7	10	11	9	8	12	9	11	12	10
	Abundance	32	30	28	23	19	23	33	41	22	18	315	139	419	355	701	396	636	287	212	179	371	608	331	2943	278	710	541	588	344	395
	QBS	43	43	33	42	42	32	46	52	65	52	108	98	113	77	98	83	112	118	77	77	77	89	98	78	78	152	91	141	105	126

(b)

		Row										Interrow										Control									
		I	II	III	IV	V	VI	VII	VIII	IX	X	I	II	III	IV	V	VI	VII	VIII	IX	X	I	II	III	IV	V	VI	VII	VIII	IX	X
Arachnida	Acarina	60	37	15	1089	44	8	16	4	3	199	143	192	717	699	181	516	1020	319	511	327	536	233	1117	255	466	1931	1394	1102	726	356
	Araneae	7	2	6	3	0	1	0	1	3	0	1	4	0	3	2	0	2	0	0	6	13	10	12	4	1	1	3	0	8	6
	Opiliones	0	0	0	0	0	0	0	0	0	0	2	0	0	1	0	0	0	0	0	0	0	0	0	0	0	0	0	0	0	0
	Pseudoscorpionida	0	0	0	0	0	0	0	0	0	0	0	0	0	0	0	0	0	0	0	0	0	0	0	0	0	0	0	0	0	0
Chilopoda	Geophilomorpha	0	0	0	0	0	0	0	0	0	0	0	0	0	0	0	0	0	0	0	0	0	0	0	0	0	0	0	0	0	0
	Lithobiomorpha	0	0	0	0	0	0	0	0	0	0	0	4	1	3	0	0	2	2	9	0	3	1	20	4	0	28	1	4	2	7
Diplopoda	Julida	0	0	0	0	0	0	0	0	0	0	0	0	0	0	0	1	0	0	0	0	0	0	0	0	0	0	0	0	1	0
	Polixenida	0	0	0	0	0	0	0	0	0	0	0	0	0	0	0	0	0	0	0	0	0	0	0	0	0	0	0	0	0	0
Pauropoda	Tetramerocerata	0	0	0	0	0	0	0	0	0	0	0	0	0	0	0	0	0	0	0	0	0	0	0	0	0	0	0	2	0	1
Symphyla	Symphyla	0	0	0	1	0	0	0	0	0	4	7	4	1	0	2	3	0	1	0	0	3	9	24	0	3	1	1	8	5	1
Malacostraca	Isopoda	0	0	0	0	0	0	0	0	0	0	0	0	0	0	0	0	0	0	0	0	1	0	1	0	0	5	1	0	0	1
Entognatha	Collembola	14	10	4	51	6	2	2	4	0	28	188	108	104	107	1672	170	246	269	219	856	62	214	1147	91	108	703	306	1262	129	461
	Diplura	0	0	0	0	0	0	0	0	0	0	0	0	0	0	0	1	0	0	0	0	0	0	0	0	0	1	0	0	2	2
	Protura	0	0	0	0	0	0	0	1	0	0	0	0	0	0	0	0	1	0	0	0	0	0	0	0	0	0	0	0	0	0
Insecta	Blattodea	0	0	0	0	0	0	0	0	0	0	0	0	0	0	0	0	0	0	0	0	0	0	0	0	0	0	0	0	0	0
	Coleoptera	0	1	4	1	0	0	0	0	0	0	3	3	4	3	1	2	3	0	2	6	2	2	2	2	2	7	0	0	34	1
	Coleoptera larvae	0	0	0	0	0	0	0	0	1	0	7	1	7	0	1	3	7	2	4	9	4	3	8	4	5	0	10	2	4	2
	Dermaptera	0	0	0	0	0	0	0	0	0	0	0	0	0	0	0	0	0	0	0	0	0	0	0	0	0	0	0	0	0	0
	Diptera	1	2	5	2	0	1	2	0	1	1	6	1	3	3	4	0	6	7	0	6	1	3	5	3	2	3	3	3	1	2
	Diptera larvae	0	0	0	1	0	0	0	0	1	1	1	1	0	0	14	0	9	1	8	8	7	3	2	0	2	0	0	6	1	4
	Hemiptera	1	5	1	0	3	0	0	0	0	3	0	2	2	9	3	23	10	50	102	6	39	8	22	28	1	15	1	37	0	102
	Hymenoptera	1	3	0	1	3	0	0	0	0	1	14	2	1	57	43	2	29	56	60	2	10	117	4	104	0	22	2	1	15	8
	Hymenoptera larvae	0	0	0	0	0	0	0	0	0	0	0	11	0	5	3	0	3	0	1	1	0	1	1	1	1	13	0	11	1	2
	Lepidoptera larvae	0	0	0	0	0	0	0	0	0	0	0	0	0	0	0	0	0	0	0	0	1	0	0	1	0	0	0	0	0	0
	Orthoptera	0	0	0	0	0	0	0	0	0	0	1	0	0	0	0	0	0	0	0	0	0	0	0	0	0	0	0	0	0	0
	Psocoptera	0	0	1	0	0	0	0	0	0	0	2	0	0	0	0	0	0	0	0	0	0	0	0	0	0	0	0	0	0	0
	Thysanoptera	0	2	0	0	3	0	0	0	0	0	0	0	0	2	1	2	2	0	0	1	2	0	5	1	0	0	0	0	0	0
	N° of groups	6	8	7	8	5	4	3	4	5	7	12	12	9	11	12	10	13	9	9	11	14	12	14	12	10	12	10	11	13	15
	Abundance	84	62	36	1149	59	12	20	10	9	237	375	333	840	892	1927	723	1340	707	916	1228	684	604	2370	498	591	2730	1722	2438	929	956
	QBS	52	54	56	87	37	34	41	61	41	73	133	132	93	103	123	98	124	107	117	98	143	131	134	113	98	152	112	137	162	182

Table S2. Abundance of arthropods groups in each condition, at **(a)** Spring and **(b)** Autumn, within Sissa GMPV system.

(a)

		Row										Interrow										Control									
		I	II	III	IV	V	VI	VII	VIII	IX	X	I	II	III	IV	V	VI	VII	VIII	IX	X	I	II	III	IV	V	VI	VII	VIII	IX	X
Arachnida	Acarina	31	9	218	95	91	64	384	127	353	100	78	64	148	98	102	123	121	97	69	39	137	112	132	131	87	101	173	229	127	471
	Araneae	0	0	0	1	0	2	0	0	1	0	2	1	0	2	0	1	5	1	2	1	1	6	1	0	0	0	3	1	2	0
	Opiliones	0	0	0	0	1	3	0	0	0	0	0	0	0	0	0	0	0	0	0	0	0	0	0	0	0	0	0	2	0	0
	Pseudoscorpionida	0	0	0	1	0	2	10	0	2	0	0	0	0	0	0	0	0	0	1	0	0	0	0	0	0	0	0	0	0	0
Chilopoda	Geophilomorpha	0	0	0	0	0	0	0	0	0	0	0	0	1	0	1	0	2	0	0	0	0	0	0	0	0	0	0	0	0	0
	Lithobiomorpha	1	0	6	0	0	0	10	0	0	0	0	1	0	0	0	0	1	0	0	0	0	0	0	0	0	0	0	0	0	0
Diplopoda	Julida	0	0	0	0	0	0	0	0	0	0	0	0	0	0	0	0	1	0	0	0	0	0	0	0	0	1	0	0	0	0
	Polixenida	1	0	0	0	0	0	0	0	0	0	0	0	0	0	0	0	0	0	0	0	0	0	0	0	0	0	0	0	0	0
Pauropoda	Tetramerocerata	0	0	0	0	0	0	0	0	0	0	0	0	0	0	0	0	0	0	0	0	0	0	0	0	0	0	0	0	0	0
Symphyla	Symphyla	0	0	0	0	0	0	0	0	0	0	0	0	0	0	0	0	0	0	0	0	0	0	0	0	0	0	0	0	0	0
Malacostraca	Isopoda	17	0	12	8	9	12	19	7	17	17	0	0	1	8	1	0	19	23	2	1	7	4	4	0	0	93	5	5	4	1
Entognatha	Collembola	35	0	24	40	11	28	61	18	45	17	23	12	24	13	13	8	58	26	13	17	10	31	7	21	27	3	60	13	14	8
	Diplura	0	0	0	0	0	0	0	0	0	0	0	0	4	0	0	0	0	0	0	0	0	0	0	0	0	1	0	0	0	0
	Protura	0	0	2	0	0	0	0	0	0	0	0	0	0	0	0	0	0	4	0	1	0	0	0	0	0	0	0	0	0	0
Insecta	Blattodea	1	0	0	0	0	1	0	0	0	0	0	0	0	0	0	0	0	0	0	0	0	0	0	0	0	0	0	1	0	0
	Coleoptera	0	0	2	2	0	1	2	0	3	2	0	1	2	1	0	0	19	0	0	0	2	5	3	0	0	1	3	0	2	1
	Coleoptera larvae	4	0	1	1	0	5	7	3	2	0	0	1	8	4	6	0	7	4	3	0	0	4	11	2	4	0	9	2	1	1
	Dermaptera	0	0	0	0	0	0	0	0	0	0	0	0	0	0	0	0	0	0	0	0	0	0	0	0	0	0	0	0	0	0
	Diptera	0	4	4	2	4	3	2	2	12	3	4	0	4	6	0	3	16	8	0	4	12	0	1	9	8	5	1	7	2	4
	Diptera larvae	0	0	0	0	0	0	0	0	0	0	0	0	1	2	0	0	0	0	0	4	0	0	2	4	0	0	0	2	9	3
	Hemiptera	0	0	0	0	0	26	0	0	0	0	39	24	2	9	0	1	20	2	19	44	0	4	10	6	1	0	2	67	60	1
	Hymenoptera	0	1	15	4	2	11	1	65	15	22	95	73	9	11	12	1	25	56	293	112	66	15	266	46	19	10	21	93	67	13
	Hymenoptera larvae	0	0	0	0	0	0	0	0	0	0	0	0	0	0	0	0	0	0	0	0	0	0	0	0	0	0	0	0	0	0
	Lepidoptera larvae	0	0	0	0	0	0	0	0	0	0	0	0	0	0	0	0	0	0	0	0	0	0	0	0	0	0	0	0	0	0
	Orthoptera	0	0	3	0	0	0	0	1	0	0	0	0	0	0	0	0	0	0	0	0	0	0	0	1	0	0	0	0	0	0
	Psocoptera	3	0	2	0	0	1	2	0	0	0	0	0	0	5	1	0	3	2	0	0	0	0	0	0	0	0	1	1	0	0
	Thysanoptera	2	0	8	6	7	0	7	1	0	4	2	0	0	5	5	4	17	3	14	2	0	6	14	0	2	8	5	4	6	12
	N° of groups	9	3	12	10	7	13	11	8	9	7	7	8	11	12	8	7	14	11	9	10	7	9	11	8	7	9	11	13	11	10
	Abundance	95	14	297	160	125	159	505	224	450	165	243	177	204	164	141	141	314	226	416	225	235	187	451	220	148	223	283	427	294	515
	QBS	97	26	99	89	67	134	109	87	88	58	78	72	107	80	96	58	94	99	92	92	58	73	88	87	58	68	75	95	104	79

(b)		Row										Interrow										Control									
		I	II	III	IV	V	VI	VII	VIII	IX	X	I	II	III	IV	V	VI	VII	VIII	IX	X	I	II	III	IV	V	VI	VII	VIII	IX	X
Arachnida	Acarina	56	69	8	27	86	70	66	52	80	145	97	422	479	50	81	178	62	61	81	19	231	289	93	10<br>3	249	28	365	306	116	65
	Araneae	1	0	0	0	1	1	1	0	2	0	0	1	2	0	0	0	0	1	0	0	0	0	0	0	2	1	0	0	0	0
	Opiliones	0	0	0	0	0	0	0	0	0	0	0	0	0	0	0	0	0	0	0	0	0	0	0	0	0	0	0	0	0	0
	Pseudoscorpionida	0	1	0	2	0	0	0	0	1	0	0	0	2	0	0	0	0	0	0	0	0	0	0	0	0	0	0	0	0	0
Chilopoda	Geophilomorpha	0	0	0	0	0	0	0	0	0	0	0	0	1	0	0	1	0	1	0	0	0	0	1	0	0	0	0	0	0	0
	Lithobiomorpha	0	2	0	2	1	2	1	1	0	3	0	1	0	0	0	1	0	2	0	0	0	0	0	0	0	0	0	0	0	0
Diplopoda	Julida	0	0	0	0	0	5	1	0	0	0	1	0	0	0	0	0	0	0	0	0	0	0	0	0	0	0	0	0	0	0
	Polixenida	0	0	0	0	0	2	0	0	0	0	0	0	0	0	0	0	0	0	1	0	0	0	0	0	0	0	0	0	0	0
Pauropoda	Tetramerocerata	0	0	0	0	0	0	0	0	0	0	0	0	0	0	0	0	0	0	0	0	0	0	0	0	0	0	0	0	0	0
Symphyla	Symphyla	0	0	0	0	1	1	0	0	0	0	0	0	0	1	0	0	0	0	0	0	0	0	0	0	0	0	0	0	0	0
Malacostraca	Isopoda	1	0	0	3	2	2	1	1	0	1	0	0	0	0	1	0	6	0	5	1	0	1	10	1	0	0	0	0	0	0
Entognatha	Collembola	7	27	0	5	54	39	26	19	33	47	121	481	144	44	32	76	32	88	33	4	107	103	84	37	98	199	249	168	51	29
	Diplura	0	0	0	0	3	1	2	1	0	14	1	0	2	1	0	3	4	0	0	0	4	0	0	3	0	1	0	0	0	0
	Protura	0	0	0	0	0	0	0	0	0	0	0	0	0	0	1	0	0	0	0	3	0	0	0	0	0	0	0	0	0	0
Insecta	Blattodea	0	0	0	0	0	0	0	0	0	0	0	0	0	0	0	0	0	0	0	0	0	0	0	0	0	0	0	0	0	0
	Coleoptera	0	3	0	0	0	2	1	0	1	6	2	0	2	1	0	0	2	0	0	0	0	4	0	0	0	0	8	0	1	1
	Coleoptera larvae	1	0	1	0	6	5	2	1	3	3	0	9	4	4	1	5	4	2	2	7	1	7	7	6	7	6	7	5	1	1
	Dermaptera	0	0	0	0	0	0	0	0	0	0	0	0	0	0	0	0	0	0	0	0	0	0	0	0	0	0	0	0	0	0
	Diptera	0	0	2	0	0	0	0	0	0	2	2	1	0	0	0	0	0	0	1	1	0	0	0	0	0	0	0	1	0	0
	Diptera larvae	0	0	0	0	0	0	0	0	0	1	0	0	1	0	0	3	0	0	0	0	0	0	0	4	0	2	0	1	0	0
	Hemiptera	0	0	0	0	16	8	4	0	5	3	3	13	0	0	0	18	16	22	62	0	0	0	0	11	2	3	2	2	1	0
	Hymenoptera	0	0	1	5	3	0	1	0	0	2	1	3	0	1	0	8	275	6	124	1	0	3	7	3	1	2	0	3	1	1
	Hymenoptera larvae	0	0	0	0	0	0	0	0	0	0	0	0	0	0	0	0	0	0	0	0	0	0	0	0	0	0	0	0	0	0
	Lepidoptera larvae	0	0	0	0	0	0	0	0	0	0	0	0	0	0	0	0	0	0	0	0	0	0	0	0	0	0	0	0	0	0
	Orthoptera	0	0	0	0	0	0	0	0	0	0	0	0	0	0	0	0	0	0	0	0	0	0	0	0	0	0	0	0	0	0
	Psocoptera	0	0	0	0	0	0	0	0	0	0	0	0	0	0	0	0	0	0	0	0	0	0	0	0	0	0	0	0	0	0
	Thysanoptera	0	0	1	1	0	0	0	1	0	0	0	0	1	0	1	0	4	1	0	1	0	1	0	0	0	0	1	4	0	0
	N° of groups	5	5	5	7	10	12	11	7	7	11	8	8	10	7	6	9	9	9	8	8	4	7	6	8	6	8	6	8	6	5
	Abundance	66	102	13	45	173	138	106	76	125	227	228	931	638	102	117	293	405	184	309	37	343	408	202	16<br>8	359	242	632	490	171	97
	QBS	61	71	37	116	137	123	89	91	77	108	88	88	97	96	81	86	88	82	67	77	60	68	75	87	57	81	53	68	57	56
